# Iron alters cell survival in a mitochondria-dependent pathway in ovarian cancer cells

**DOI:** 10.1042/BJ20140878

**Published:** 2015-02-20

**Authors:** Kyle Bauckman, Edward Haller, Nicholas Taran, Stephanie Rockfield, Abigail Ruiz-Rivera, Meera Nanjundan

**Affiliations:** *Cancer Biology Ph.D Program, Moffitt Cancer Center and Research Institute, Tampa, FL 33612, U.S.A.; †Department of Integrative Biology, University of South Florida, Tampa, FL 33620, U.S.A.; ‡Department of Cell Biology, Microbiology, and Molecular Biology, University of South Florida, Tampa, FL 33620, U.S.A.

**Keywords:** aconitase, ferric ammonium citrate (FAC), glutamate, GSH, holo-transferrin (HTF), iron, MAPK, mitochondria, mitochondrial calcium uniporter, reactive oxygen species, Ru360, TOM20, TOM70, FAC, ferric ammonium citrate, FTH, ferritin, GAPDH, glyceraldehyde-3-phosphate dehydrogenase, GSH, glutathione, HTF, holo-transferrin, H_2_DCFDA, 2′,7′-dichlorodihydrofluorescein diacetate, H_2_O_2_, hydrogen peroxide, IMM, inner mitochondrial membrane, IRE, iron-responsive element, IRP, iron regulatory protein, LMP, lysosomal membrane permeabilization, MAPK, mitogen-activated protein kinase, MCU, mitochondrial calcium uniporter, NAC, *N*-acetyl-L-cysteine, OMA, oxalomalate, OMM, outer mitochondrial membrane, ROS, reactive oxygen species, TOM, translocase of outer membrane

## Abstract

The role of iron in the development of cancer remains unclear. We previously reported that iron reduces cell survival in a Ras/mitogen-activated protein kinase (MAPK)-dependent manner in ovarian cells; however, the underlying downstream pathway leading to reduced survival was unclear. Although levels of intracellular iron, ferritin/CD71 protein and reactive oxygen species did not correlate with iron-induced cell survival changes, we identified mitochondrial damage (via TEM) and reduced expression of outer mitochondrial membrane proteins (translocase of outer membrane: TOM20 and TOM70) in cell lines sensitive to iron. Interestingly, Ru360 (an inhibitor of the mitochondrial calcium uniporter) reversed mitochondrial changes and restored cell survival in HEY ovarian carcinoma cells treated with iron. Further, cells treated with Ru360 and iron also had reduced autophagic punctae with increased lysosomal numbers, implying cross-talk between these compartments. Mitochondrial changes were dependent on activation of the Ras/MAPK pathway since treatment with a MAPK inhibitor restored expression of TOM20/TOM70 proteins. Although glutathione antioxidant levels were reduced in HEY treated with iron, extracellular glutamate levels were unaltered. Strikingly, oxalomalate (inhibitor of aconitase, involved in glutamate production) reversed iron-induced responses in a similar manner to Ru360. Collectively, our results implicate iron in modulating cell survival in a mitochondria-dependent manner in ovarian cancer cells.

## Abstract 2

Non-transferrin bound iron mediates cell death in ovarian cancer cells by inducing mitochondrial damage and loss of OMM proteins. Inhibition of the mitochondrial calcium uniporter, MAPK and aconitase activity reversed these changes.

## INTRODUCTION

Iron is an essential co-factor for enzymes which regulate multiple cell functions, including survival [1–3]. Iron, obtained from diet sources or from heme, binds to transferrin (in blood) which shuttles this nutrient as diferric–transferrin [i.e. holo-transferrin (HTF)] complex [1–3]. It is recognized by the transferrin receptor (CD71) and undergoes receptor-mediated endocytosis [1–3]. Iron, present as Fe^+3^, is reduced to its active Fe^+2^ form prior to its export into the cytosolic compartment; this increases the intracellular concentration of iron in the labile iron pool [1–3]. When iron is in excess, it binds to ferritin (FTH) (induced under high iron levels) for storage, whereas CD71 levels are reduced, leading to a regulated mechanism to control intracellular iron levels [1–3]. This mechanism of regulation occurs primarily via the action of iron regulatory protein (IRP)1/2 which binds to regulatory elements in the 3′-untranslated or 5′-untranslated regions of FTH and CD71 mRNA, respectively [1–3]. When intracellular iron levels are low, iron is mobilized from FTH-bound iron stores via entry into the autophagosomal–lysosomal pathway, leading to the release of Fe^+2^ in a process called ferritinophagy [4]. The excess iron can also be exported extracellularly via ferroportin, a plasma membrane localized channel whose levels are regulated by hepcidin (a small peptide hormone which promotes the degradation of ferroportin and increases intracellular retention of iron) [5].

Although increased intracellular iron levels can promote proliferation [6], we [7] and others [8] have found that iron can promote cell death [7,9]; however, the mechanism through which iron elicits these effects remains unclear. We recently reported that the mitogen-activated protein kinase (MAPK) pathway reduces cell survival in response to iron presented as ferric ammonium citrate (FAC, non-transferrin bound iron often found in iron overload conditions such as in endometriotic cysts [10]) in ovarian cell types associated with Ras mutations or overexpression [7]. In particular, the HEY serous ovarian carcinoma cell line succumbed to an apoptotic/necrotic response when exposed to FAC; cellular changes included those in the autophagic/lysosome pathway which could be reversed by inhibition of MAPK [7].

Since mitochondrial changes were identified in ferroptosis (a novel iron-regulated cell death pathway) [11] and mitochondria import iron for various functions (i.e. regulation of the electron transport chain, heme synthesis and iron–sulfur cluster biogenesis) [12,13], our goal in the present study was to determine whether mitochondrial changes were present in iron-treated HEY ovarian carcinoma cells. Although we did not observe any marked differences in expression of proteins involved in cellular iron transport or metabolism (i.e. FTH and CD71), we noted that the outer mitochondrial transporter proteins, translocase of outer membrane 20 (TOM20) and TOM70, were markedly reduced in HEY cells; furthermore, these changes could be reversed with U0126 (MAPK inhibitor). By TEM, we identified damaged mitochondria and the process of mitophagy. TOM20 and TOM70 protein levels could be reversed by cellular treatment with Ru360, an inhibitor of the mitochondrial calcium uniporter (MCU) [14,15]. Ru360 also reversed induction of autophagy as well as markedly increasing lysosome numbers, suggesting that inhibition of the uniporter may lead to mitophagy as well as lysosomal loss [via lysosomal membrane permeabilization (LMP)]. This result implys cross-talk between the mitochondrial, autophagic and lysosomal compartments. Moreover, we noted that cellular treatment with oxalomalate (OMA), an inhibitor of aconitase activity (which targets both cytosolic and mitochondrial forms), partly reversed the effect of iron (cell death and changes in TOM20/TOM70), although extracellular glutamate levels were unchanged (relative to untreated cells) in response to iron. Collectively, these results indicate that mitochondrial damage induced by FAC lies downstream of MAPK activation; these mitochondrial events are linked to lysosomal and autophagy events which contribute to iron-induced cell death in the HEY ovarian cancer cells.

## EXPERIMENTAL

### Cell lines

HEY ovarian carcinoma cells, immortalized (LTAg/hTERT) normal ovarian surface epithelial cells (T80), and T80 cells overexpressing H-Ras were kindly provided by Dr. Gordon Mills (MD Anderson Cancer Center); these cells were maintained in RPMI 1640 in 8% FBS and penicillin/streptomycin. TOV112D endometrioid ovarian carcinoma cells (ATCC) and TOV21G clear cell ovarian carcinoma cells (kindly provided by Dr. Johnathan Lancaster, Moffitt Cancer Center, Tampa, FL) were maintained in MCDB131:Medium 199 (1:1 ratio) supplemented with 8% FBS and penicillin/streptomycin. All cell lines were tested to be mycoplasma negative and were STR profiled (Genetica Laboratories) prior to use in the studies reported herein.

### Cellular treatments with FAC and/or inhibitors

FAC (Fisher Scientific) was dissolved in tissue culture-grade PBS and utilized at a final concentration of 100 or 250 μM. We have used FAC with treatment times of between 1, 6, 18, 24 and 48 h in the various Figures depending on the functional outcome assessed. U0126 was dissolved in DMSO (Cell Signaling Technology) and used at a final dose of 10 μM. Ru360 (Fisher Scientific) was dissolved in de-oxygenated tissue-culture grade water and utilized at a final concentration of 10 μM. OMA (sodium salt) (Cayman Chemicals) was dissolved in PBS and used at a final concentration of 5 mM. Hydrogen peroxide (H_2_O_2_; Fisher Scientific) was used at a final concentration of 100 μM. HTF (R&D Systems) was dissolved in PBS and utilized at concentrations from 0.0001 μg/ml to 100 mg/ml.

### Cellular treatments with siRNA

T80 cells were seeded at 325000 cells/well in a six-well plate. Following overnight adherence, cells were transfected with siRNA targeting IRP1 (ACO1, L-010037-01-0005), IRP2 (IREB2, L-022281-01-0005), TOM20 (TOMM20, L-006487-01-0005), TOM70 (TOMM70, L-021243-01-0005) or non-targeting ON-TARGETplus control (D-001810-10-0005) with Dharmafect I (ThermoScientific) using methods previously described [7].

### RNA isolation and real-time PCR

Total RNA was isolated using the RNeasy Mini Kit (Qiagen) as previously described [17]. Hepcidin (Hs00221783_m1) and One-step Master Mix (Applied Biosystems) were utilized to perform real-time PCR using previously described methodology [7]. Results were analysed by the comparative method using untreated cells as the reference sample and β-actin for normalization. RNA-fold changes were calculated using the following formula: 2^−ΔΔCT^.

### Protein harvest, SDS/PAGE, and Western analyses

Protein isolation and Western blot analyses were performed according to previously published methods [7]. Primary antibodies were obtained from the following sources and used at the following dilutions: LC3B rabbit polyclonal (#2775S, 1:1000), p-ERK1/2 rabbit polyclonal (#9101S, 1:1000), total MAPK rabbit polyclonal (#4695S, 1:1000), glyceraldehyde-3-phosphate dehydrogenase (GAPDH) rabbit polyclonal (#2118S, 1:4000), and FTH1 rabbit polyclonal (#3998S, 1:1000) were obtained from Cell Signaling Technology. CD71 mouse monoclonal (2B6, sc-51829, 1:100), TOM20 rabbit polyclonal (FL-145, sc-11415, 1:7500), TOM70 mouse monoclonal (A-8, sc-390545, 1:1000), IRP1 goat polyclonal (N-17, sc-14216, 1:1000) and IRP2 mouse monoclonal (7H6, sc-33682, 1:500) were obtained from Santa Cruz Biotechnology.

### Quantification of intracellular iron levels

Iron was measured using a commercially available kit (ABNOVA) according to the manufacturer's instructions. Briefly, cells were grown in 100 mm^2^ dishes and treated with FAC. At the time of measurement, cells were trypsinized and washed extensively in PBS. Cells were then lysed into 100 μl of iron assay buffer followed by centrifugation at 20817 *g* for 10 min. Fifty microlitres of sample was added to each well of a 96-well plate followed by addition of 50 μl of iron assay buffer. Iron reducing agent (5 μl) was added to both samples and standards (0–10 nmol/well in a total volume of 100 μl) followed by incubation at room temperature for 30 min. This was then followed by the addition of 100 μl of iron probe and mixing on a BioTek Synergy 2 plate reader (BioTek Instruments Inc.) for 1 min. The plate was incubated for a further 1 h (protected from light) and then read on the Biotek plate reader at 593 nm.

### Quantification of reactive oxygen species

Cells were seeded in black 96-well plates and allowed to adhere following overnight incubation. The cell-permeant 2′,7′-dichlorodihydrofluorescein diacetate (H_2_DCFDA) dye (Life Technologies) was added to a final concentration of 10 μM in warm PBS after discarding the medium from the 96-well plate. The cells were loaded with dye by incubating the plate at 37°C for 30 min. The PBS-dye solution was then discarded and then the cells were treated with 250 μM FAC or 100 μM H_2_O_2_ (as positive control) for between 6 and 24 h. The plate was read on a Biotek plate reader using an excitation wavelength of 495 nm (filter 485/20) and an emission wavelength of 529 nm (filter 530/20).

For measurement of reactive oxygen species (ROS) via flow cytometry, cells were seeded in 6-well plates at 250000 cells/well. Following overnight attachment, the H_2_DCFDA dye was added to a final concentration of 10 μM in warm PBS and handled as described above. FAC (or H_2_O_2_) treatment was performed for 24 h. Both culture supernatant and adherent cells (collected via trypsinization) were centrifuged at 106 *g* for 5 min and resuspended in 500 μl of PBS for analysis by flow cytometry (Karoly Szekeres, Flow Cytometry Core Facility, College of Medicine, University of South Florida).

### Lysosome staining with LysoTracker Red

Lysosome staining with LysoTracker Red was carried out according to previously published methods [7]. Briefly, cells were seeded on to glass coverslips, allowed to adhere and then treated with 250 μM FAC for 24 h. LysoTracker Red (Life Technologies) was added (75 nM) 1 h prior to completion of FAC treatment (24 h). Cells were washed in PBS and blocked for 1 h (5% goat serum in PBS containing 0.1% Triton X-100). This was followed by three PBS washes, addition of DAPI/antifade solution, and mounting on to glass slides. Slides were viewed and imaged using a PerkinElmer UltraVIEW Confocal spinning disc microscope (PerkinElmer Corporation).

### TEM

Matched cultures of control and experimental HEY cells grown to confluence, exposed to FAC at 6, 18 and 24 h, were submitted for TEM. The methods for TEM have been described previously [7].

### Direct immunofluorescence

For direct immunofluorescence microscopy, cells were seeded on to coverslips and allowed to adhere following overnight incubation. Cells were transfected with EGFP-LC3 (#11546; Addgene) [16] using methods previously reported and prepared for direct immunofluorescence according to previously published methods [7]. The cells were viewed using a PerkinElmer UltraVIEW Confocal spinning disc microscope (PerkinElmer Corporation). Quantification of EGFP-LC3 expressing cells was performed by counting the number of cells expressing a punctate pattern (at least 20 punctae) in a total of 200 EGFP-LC3 positive cells.

### Quantification of intracellular glutathione levels

Cells were seeded into white opaque 96-well plates and, following overnight adherence, were treated with 250 μM FAC for 24 h. The assay was performed using previously published methods (Promega) [17]. The medium was discarded, cells washed with PBS and then GSH-glo added (100 μl) to all wells containing sample. The plate was mixed using a BioTek Synergy 2 plate reader (BioTek Instruments Inc.) for 2 min followed by room temperature incubation for 30 min. This was followed by addition of 100 μl of luciferin reagent, followed by 15 min incubation at room temperature. The samples were read on a Biotek plate reader to detect luminescence.

### Quantification of intracellular glutamate levels

Glutamate levels were measured using a commercially available kit (Sigma–Aldrich) according to the manufacturer's instructions. Briefly, cells were grown in six-well plates and treated with FAC for 24–48 h. At the time of measurement, culture medium supernatant was collected and centrifuged at 20817 *g* for 1 min (to sediment any cellular material). Twenty-five microlitres of each sample (as well as standards) was added to a clear 96-well plate followed by the addition of 25 μl of glutamate assay buffer. The plate was incubated for 30 min at 37°C and then read on a Biotek plate reader at 450 nm. The results are reported as the total glutamate concentration in the sample [based on the standards and sample volume added to wells (in mM)].

### Statistical analyses

Experimental replicates were conducted as indicated in each Figure legend presented. Error bars displayed on the bar graphs represent SDs. *P* values were calculated using Graphpad Prism 6.04 (standard Student's *t*-test). NS refers to not significant *P* values (*P* > 0.05). * refers to *P*<0.05, ** refers to *P*<0.01, *** refers to *P* < 0.001, and **** refers to *P* < 0.0001.

## RESULTS

### Iron increases ROS in multiple ovarian cell lines while reducing glutathione levels in the iron-sensitive HEY cells

**Figure 1:**
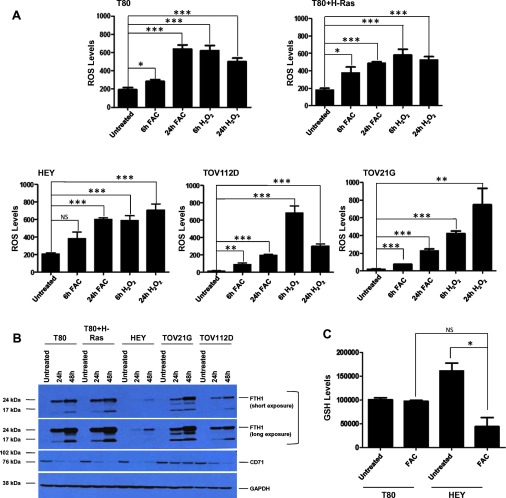
Iron increases ROS in multiple ovarian cell lines, whereas GSH levels diminish upon iron treatment in HEY cells (**A**) T80, T80+H-Ras, HEY, TOV112D and TOV21G cells were assessed for ROS as described in the Experimental section (*n*=2). (**B**) T80, T80+H-Ras, HEY, TOV112D and TOV21G cells were treated with 250 μM FAC for 24 and 48 h. Cell lysates were harvested and analysed via Western blotting using the following antibodies: (1) FTH1, (2) CD71, and GAPDH. (*n*=3). (**C**) T80 and HEY cells were assessed for GSH levels as described in the Experimental section (*n*=3).

Since ROS are frequently associated with cellular stress [18], we assessed whether iron can increase ROS which may then lead to the observed functional consequence of reduced cellular viability we reported earlier [7]. To measure ROS, we utilized the H_2_DCFDA dye, which is a general oxidative stress indicator allowing measurement of H_2_O_2_ as well as superoxide anion [19]. We assessed ROS levels across multiple ovarian cell types including T80 (immortalized normal ovarian surface epithelial cell line), T80+H-Ras (T80 cells overexpressing H-Ras), HEY (serous epithelial ovarian carcinoma cell line), TOV112D (endometrioid ovarian carcinoma cell line) and TOV21G (clear cell ovarian carcinoma cell line) which were treated with 250 μM FAC. This dose of FAC (non-transferrin-bound iron frequently elevated in blood from patients with iron-overload conditions [10]) was selected based on iron-overload conditions present in endometriotic cysts, which are reported to have mM levels of free iron [20–23] and in which endometriotic epithelial cells are considered the precursor lesions which may transition to ovarian cancer [20–23]. As a positive control, we treated these five cell lines with 100 μM H_2_O_2_ which can induce an increase in intracellular ROS species [19]. As shown in Figure 1A, all cell lines displayed increased ROS with up to 24 h FAC treatment. In addition, H_2_O_2_ treatment, itself, resulted in a significant increase in ROS up to 24 h following treatment. Since the ROS levels in the FAC-sensitive HEY, TOV21G, and T80+H-Ras cell lines were not markedly different from the FAC-resistant cell lines (T80 and TOV112D), these results indicate that the ROS levels following FAC treatment do not correlate with the iron-induced reduction in cell viability. Furthermore, cellular treatment of FAC in combination with *N*-acetyl-L-cysteine (NAC) or catalase (antioxidants/ROS scavengers) did not reverse the cell death response associated with FAC in HEY cells (results not shown). In addition, we assessed whether the expression levels of FTH1 and CD71 could account for the FAC-mediated reduction in cellular viability in FAC-sensitive cell lines (T80+H-Ras, HEY, and TOV21G). As shown in Figure 1B, we did not identify any correlation in FTH induction or CD71 down-regulation across these cell lines in the absence or presence of FAC treatment (24 or 48 h). Therefore, another mechanism is likely responsible for the FAC-mediated reduction in cellular viability in FAC-sensitive cell lines.

To determine whether glutathione (GSH) levels were altered by FAC treatment in both iron-sensitive and iron-resistant cell lines, we treated T80 and HEY cells with 250 μM FAC and then quantified GSH levels. As shown in Figure 1C, GSH levels were not altered in the T80 cell line upon FAC treatment (relative to untreated cells), whereas they were significantly reduced in the iron-sensitive cell line, HEY. We also assessed changes in GSH levels following FAC treatment in T80+H-Ras, TOV21G and TOV112D cells; however, GSH levels were variable in these cell lines (results not shown). Nonetheless, these results suggest that the reduction in the antioxidant GSH levels may contribute to the iron-induced apoptotic/necrotic response in iron-sensitive HEY cells.

### Contribution of IRP1/2 to FTH and CD71 protein expression upon FAC treatment in ovarian cancer cells

We previously assessed the basal expression level of genes involved in modulating cellular iron content, specifically FTH and CD71 in multiple gynaecological cell types [7]. Although we did not identify an association between iron sensitivity and expression level of these specific IRPs, we extended the analysis to investigate changes in expression of IRP targets following FAC treatment. Similarly, we did not identify a correlation between these specific IRPs (FTH1 and CD71) and the sensitivity of the ovarian cells to FAC (Figure 1B). Furthermore, we assessed the levels of hepcidin mRNA [5]; however, the transcript levels were undetectable via real-time PCR (cycle threshold >40). We also quantified relative intracellular iron levels across the ovarian cell lines; yet, we did not identify any marked differences among the iron-sensitive and resistance cell lines (results not shown). These results suggest that the expression levels of the IRP targets (and intracellular iron levels) in response to FAC do not appear to be correlated with iron-induced sensitivity.

**Figure 2:**
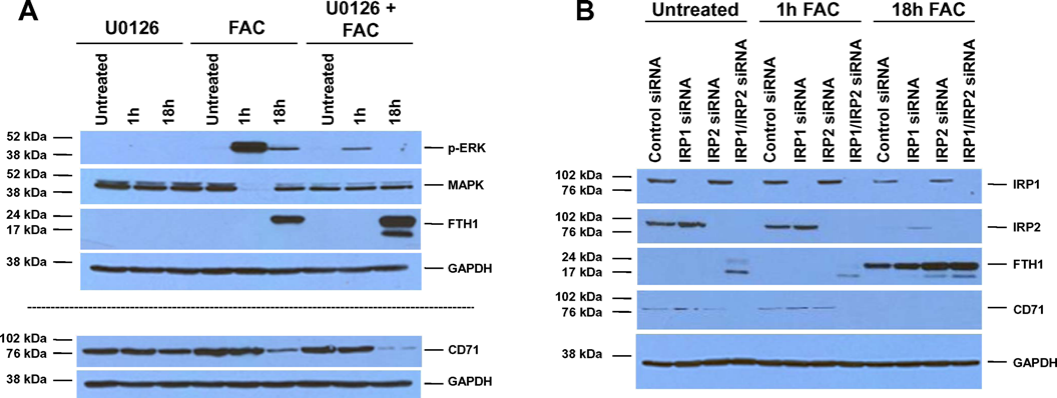
Contribution of IRP1/2 to ferritin and CD71 protein expression upon FAC treatment (**A**) HEY cells were seeded at 250000 cells in each well of a six-well plate. After overnight attachment, the cells were treated for 18 h with U0126 alone, FAC alone or U0126 and FAC combination. Cell lysates were harvested and analysed via Western blotting using the following antibodies: (1) p-ERK, (2) total MAPK, (3) ferritin (FTH1), (4) CD71 and (5) GAPDH (*n*=2). (**B**) HEY cells were seeded at 325000 cells in each well of a six-well plate. Following cellular adherence, the cells were treated with two rounds of control non-targeting, IRP1, IRP2 or IRP1 and IRP2 siRNA on sequential days as described in the Experimental section. Iron (250 μM) was then added for 1 or 18 h. Cell lysates were harvested and analysed via Western blotting using the following antibodies: (1) IRP1, (2) IRP2, (3) ferritin (FTH1), (4) CD71 and (5) GAPDH (*n*=4).

Since we previously reported that iron-induced apoptotic/necrotic response in HEY cells was mediated in a MAPK-dependent manner, we assessed whether changes in FTH and CD71 (key targets of IRP1/2) were mediated in a MAPK-dependent manner. This was performed by co-treating HEY cells with FAC and U0126, a MAPK inhibitor. As shown in Figure 2A, the combinatorial treatment did not reduce FTH or increase CD71 protein levels. In contrast, FTH protein was further increased and CD71 was further reduced, suggesting that MAPK activation antagonizes the ability of IRP1/IRP2 to regulate the translation of these two critical IRP targets.

In order to assess the contribution of IRP1 and IRP2 to the regulation of FTH and CD71 [1] following iron treatment, we performed knockdown studies in HEY cells targeting IRP1 alone, IRP2 alone, or IRP1 and IRP2 combinatorial knockdown. As shown in Figure 2B, with a knockdown efficiency of IRP1 and IRP2 greater than 90%, we noted that there was a marked reduction in CD71 protein upon knockdown of both IRP1 and IRP2 (in untreated cells and 1 h FAC treatment). Following 18 h FAC treatment, CD71 protein was dramatically reduced, whereas FTH levels were increased. In addition, we observed a marked increase in FTH levels with IRP1 and IRP2 siRNA alone (or in combination). Collectively, these results suggest that both IRP1 and IRP2 appear to oppose FAC-induced FTH production in HEY cells, implying that additional mechanisms are involved in FTH regulation.

### FAC induces mitochondrial damage and loss of OMM proteins in the iron-sensitive HEY cell line

**Figure 3:**
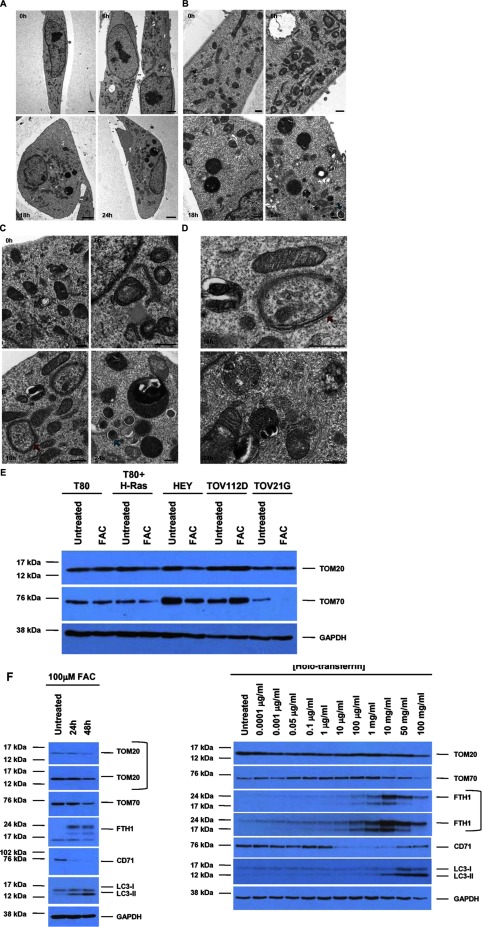
FAC induces mitochondrial damage and loss of OMM proteins in the iron sensitive HEY cell line Low (**A**) and medium (**B** and **C**), magnification TEM images are shown for HEY cells at 0, 6, 18 and 24 h FAC treatment. (**D**) High-magnification TEM images are shown for HEY cells at 18 and 24 h FAC treatment. (**A**) Low-magnification images of HEY cells, 0 time, 6, 18 and 24 h exposure to FAC. Note progressive mitochondrial changes and accumulated secondary lysosomes. Scale bar equals 2 μm. (**B**) Higher magnification images of cells at 0, 6, 18 and 24 h exposure to FAC. Cells show progressive damage to mitochondria, loss of rough endoplasmic reticulum, phagocytosis of mitochondria (blue arrow) and progressive increase in accumulation of dense secondary lysosomes. Scale bar equals 500 nm. (**C**) At 0 h, numerous mitochondria in the cytoplasm, as well as a profile of rough endoplasmic reticulum, a small lysosomal vacuole and some Golgi vesicles are shown. Cristae in these mitochondria are well ordered and the matrix of the mitochondria is dense. At 6 h, a mitochondrion undergoing degeneration, as well as several other mitochondria with disrupted, swollen cristae are shown. At 18 h, several autophagocytic membranes are seen (red arrow) surrounding portions of the cytoplasm. The cytoplasm also contains a lysosome, several mitochondria and a profile of rough endoplasmic reticulum. At 24 h, the cell contains a large digestive vacuole, along with numerous smaller ones. The cell also contains eight autophagocytic vacuoles (blue arrow) at various stages of formation, along with two mitochondria with disrupted cristae. No endoplasmic reticulum is visible in this field of view. Scale bar equals 500 nm. (**D**) 18 h treatment, HEY cell showing an enlargement of an autophagolysosome (red arrow) and a lysosome in the cytoplasm of the cell. At 24 h, the photograph shows vesicle-filled digestive vacuoles, a degenerating mitochondrion, an autophagocytic vacuole membrane and a lysosome (upper left of photograph). Scale bar equals 500 nm. (**E**) T80, T80+H-Ras, HEY, TOV112D and TOV21G cells were seeded at 250000 cells in each well of a six-well plate. Following adherence, the cells were treated with 250 μM FAC for 48 h. Cell lysates were harvested and analysed using the following antibodies: (1) TOM20, (2) TOM70 and (3) GAPDH (*n*=2). (**F**) HEY cells were treated for 24 or 48 h with 100 μM FAC (left panel) or a range of holo-transferrin (HTF) doses (0.0001 μg/ml to 100 mg/ml) (right panel). Cell lysates were collected and analysed by Western blotting using the following antibodies: (1) TOM20, (2) TOM70, (3) FTH1, (4) CD71, (5) LC3B and (6) GAPDH (*n*=2).

In order to assess structural changes in the cellular compartments following FAC treatment, we performed TEM in HEY cells treated with 250 μM FAC up to 24 h. As shown in Figures 3A–3D, untreated HEY cells consisted of cells with flattened morphology containing scattered mitochondria. These mitochondria were condensed and a subset contained a light staining matrix with calcium granules and well-defined cristae. Figure 3A is a panel of four low magnification electron micrographs of HEY cells at different time frames following exposure to FAC, untreated, 6, 18 and 24 h post-exposure. Untreated cells have flattened morphology with condense nucleolus in the nucleus and numerous mitochondria in the cell cytoplasm. At 6 h, two cells contain nuclei with enlarged nucleoli with dispersed heterochromatin. Numerous mitochondria in the cells exhibit swelling. A large vacuole is observed in the cytoplasm of one cell. Vacuolization appears in the Golgi region of a second cell. At 18 h, the cell contains multiple dark-staining digestive vacuoles and swollen mitochondria. The cell cytoplasm contains numerous swollen and degenerating mitochondria as well as large digestive vacuoles. Higher magnification images in Figures 3B and 3C show that within 6 h FAC treatment, a few mitochondria were observed to be encapsulated by autophagosomal membrane; however, most mitochondria appeared intact, along with the other organelles such as endoplasmic reticulum and Golgi. Following 18 h FAC, we noted an increase in dark coloured secondary lysosomes together with increased autophagic activity. A subset of mitochondria appeared damaged. At 24 h FAC treatment, the cytoplasm contained numerous darkly stained secondary lysosomes, small autophagosomes, and degenerating mitochondria which were in the process of being consumed by autophagosomal membrane. These results suggest that FAC induces mitochondrial damage as well as autophagic activity in HEY cells.

Since the TEM micrographs showed mitochondrial damage in response to FAC, we next assessed changes in the expression level of outer mitochondrial membrane (OMM) proteins such as TOM20 and TOM70, which are critical in maintaining OMM integrity [24,25]. As shown in Figure 3E, following FAC treatment, the expression of both mitochondrial proteins (to a greater extent with TOM70) was reduced in the HEY cell line (Ras mutated) [7]. In addition, TOM20 was reduced in T80 cells overexpressing H-Ras whereas TOV21G (which harbours a Ras mutation) [7] had a marked reduction in TOM70. Both the TOV112D and the T80 cells (lacking Ras mutations) [7] did not elicit any marked changes in the TOM proteins assessed. In Figure 3F, we assessed changes in TOM20 and TOM70 in HEY cells treated with 100 μM FAC and HTF (including a dose-response from 0.0001 μg/ml to 100 mg/ml). Consistently, we noted elevated FTH/reduced CD71 protein levels as well as changes in LC3B (an autophagy marker)) under these conditions. However, although the changes in TOM20/70 proteins were clearly noted with 100 μM FAC (in addition to reduced cell survival response), changes in these OMM proteins occurred to a lesser extent with HTF (10–50 mg/ml which is 250 μM) with no change in cellular viability (results not shown). Collectively, these results suggest that iron treatment (as a non-transferrin bound form which enters cells independently of the CD71 [10]) induces loss of specific OMM proteins in cell lines sensitive to FAC treatment.

### Inhibition of the MCU reverses FAC-mediated loss of TOM expression and cell death response in HEY cells

**Figure 4:**
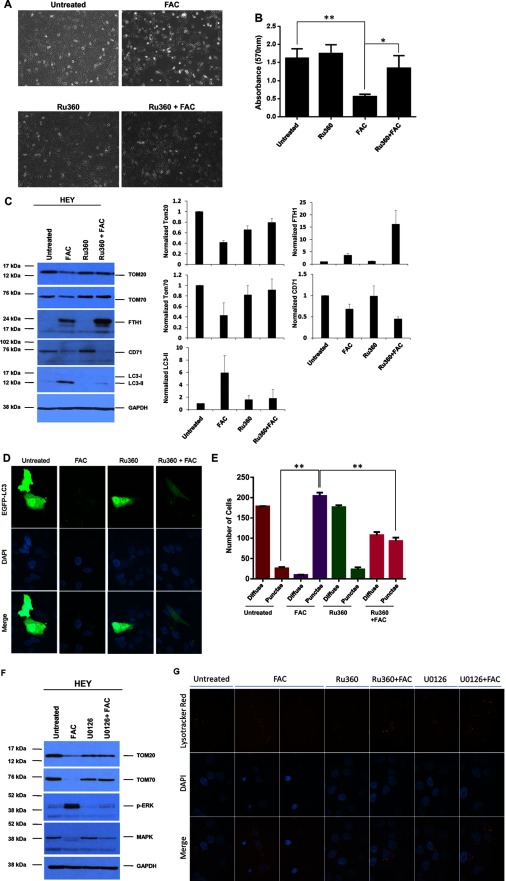
Inhibition of mitochondrial iron import reverses FAC-mediated loss of OMM proteins and cell death response in HEY cells (**A**) HEY cells were seeded at 250000 cells in each well of a six-well plate. Following adherence cells were treated with FAC (250 μM), Ru360 (10 μM) or Ru360 (10 μM) in combination with FAC (250 μM) for 48 h. Representative light microscope images were then captured. (**B**) Cells were treated with 100 μM FAC as described in (A). Cells were then stained with Crystal Violet and dissolved in Sorensons’ buffer for quantification at 570 nm (*n*=3). (**C**) Cells were treated as described in (A). Cell lysates were harvested and analysed via Western blotting (left panels) using the following antibodies: (1) TOM20, (2) TOM70, (3) FTH1, (4) CD71, (5) LC3B and (6) GAPDH (*n*=2). On the right panels, densitometric analyses of the Western blots are shown (*n*=4). (**D**) HEY cells were seeded on to glass coverslips at 250000 cells in each well of a six-well plate. Following overnight adherence, cells were transfected with pEGFP-LC3 plasmid. Cells were then treated with FAC (250 μM) alone, Ru360 (10 μM) alone or Ru360 (10 μM) in combination with FAC (250 μM). They were then viewed using a confocal microscope; representative images were acquired using a 60× oil immersion objective (*n*=4). (**E**) Quantitative data for results presented in (D) were obtained by counting at least 200 GFP positive cells and the number of cells containing more than 20 punctae (*n*=4). (**F**) HEY cells were seeded at 250000 cells in each well of a six-well plate. Following overnight adherence, cells were treated with FAC (250 μM) alone, U0126 (10 μM) alone or U0126 (10 μM) in combination with FAC (250 μM). Cell lysates were then analysed via Western blotting using the following antibodies: (1) TOM20, (2) TOM70, (3) p-ERK, (4) total MAPK and (5) GAPDH (*n*=2). (**G**) HEY cells were seeded on to glass coverslips at 250000 cells in each well of a six-well plate. Following overnight adherence, cells were as described in (F). After 24 h treatment, LysoTracker Red was added for 1 h incubation. Cells were stained with DAPI and imaged using a confocal microscope and a 60× oil immersion objective. Representative images are shown (*n*=4).

Since the MCU has been reported to be involved in iron import into mitochondria [15,26,27], we thus assessed its contribution to the FAC-mediated cell death response and mitochondrial changes in HEY cells. To address this question, we utilized Ru360, an inhibitor of the MCU [26,27]. As shown in Figures 4A and 4B, the combinatorial treatment of FAC with Ru360 increased cellular survival of HEY cells. Strikingly, the combinatorial treatment showed that the TOM20 and TOM70 protein levels, which were reduced following treatment with FAC alone, could be restored to basal levels (Figure 4C). These results implicate the involvement of the MCU in reducing the expression of OMM proteins. FTH protein was markedly more elevated while CD71 protein was decreased in cells co-treated with FAC and Ru360 relative to FAC alone (Figure 4C); these results suggest that inhibition of the MCU leads to changes in expression of IRP1 targets. Similar results were obtained with 100 μM FAC (results not shown). In addition, we noted that Ru360 in combination with FAC reversed changes in LC3-II expression (Figure 4C); indeed, this was validated using the EGFP-LC3 fluorescence-based assay to monitor autophagic flux (Figure 4D and 4E). These changes in autophagy suggest that activation of this pathway occurs downstream of the mitochondrial events.

We previously reported that FAC-mediated cellular responses in HEY cells were mediated by the MAPK pathway [7]. As shown in Figure 4F, we now demonstrate that co-treatment of FAC with U0126 (MAPK inhibitor) reversed the changes in TOM20 and TOM70 protein expression; this result implicates the MAPK pathway in mediating FAC-induced changes in OMM protein expression. Furthermore, assessment of changes in lysosome numbers via LysoTracker Red staining demonstrated that the Ru360 combinatorial treatment with FAC markedly increased lysosomal punctae, similar to the response noted with U0126 (Figure 4G and our previous report [7]). Collectively, these results suggest cross-talk between the mitochondrial and lysosomal compartments in response to FAC treatment. As shown in Supplementary Figure S1, we observed that knockdown of TOM70 led to a dramatic increase in LC3-I (and decreased autophagic punctae) as well as increased lysosome numbers (relative to non-targeting siRNA) both in the absence and presence of FAC; these results also imply cross-talk between the mitochondrial and lysosomal organelles.

To assess the contribution of the mitochondrial compartment to ROS generation following iron treatment, we measured ROS in cells that were co-treated with Ru360 and FAC. However, we did not observe any reversal of ROS (Supplementary Figure S2), suggesting that ROS is unlikely to mediate mitochondrial changes, autophagic or lysosome responses. Interestingly, we noted that Ru360, by itself, increased ROS levels in HEY cells; this could suggest that the activity of the MCU is needed to maintain low levels of ROS.

### OMA (aconitase inhibitor) reverses FAC-mediated loss of TOM expression and cell death in HEY cells

**Figure 5:**
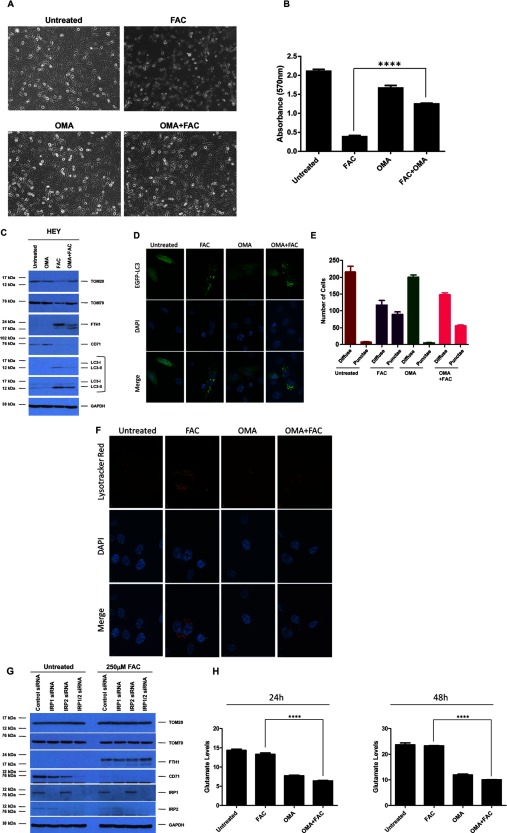
Oxalomalate reverses FAC-mediated loss of OMM proteins and cell death in HEY cells (**A**) HEY cells were seeded at 250000 cells in each well of a six-well plate. Following adherence, cells were treated with FAC (250 μM) alone, OMA (5 mM) alone or FAC (250 μM) in combination with OMA (5 mM) for 48 h. Representative light microscope images were then captured. (**B**) HEY cells were seeded at 5000 cells per well in a 96-well plate. Following attachment, cells were treated as described in (A), stained with Crystal Violet and dissolved with Sorenson's Buffer followed by quantification at 570 nm. (**C**) HEY cells were treated as described in (A). Cell lysates were analysed by Western blotting using the following antibodies: (1) TOM20, (2) TOM70, (3) FTH1, (4) CD71, (5) LC3B and (6) GAPDH (*n*=2). (**D**) HEY cells were seeded at 250000 cells in each well of a six-well plate. Following adherence, cells were transfected with pEGFP-LC3 and then treated as described in (A) for 24 h. Images were then captured using a confocal microscope and a 60× oil immersion objective lens and representative images are shown (*n*=2). (**E**) Quantitative data for results presented in (D) were obtained by counting at least 200 GFP positive cells and assessing the number of cells containing more than 20 punctae (*n*=2). (**F**) HEY cells were treated as described in (A) for 24 h. Following treatment, LysoTracker Red was added for 1 h. The cells were stained with DAPI and viewed using a confocal microscope with a 60× oil immersion objective lens. Representative images are shown (*n*=2). (**G**) Following cellular adherence, HEY cells were treated with control non-targeting, IRP1, IRP2 or IRP1 and IRP2 siRNA. Iron (250 μM) was then added for 48 h. Cell lysates were harvested and analysed via Western blotting using the following antibodies: (1) TOM20, (2) TOM70, (3) FTH1, (4) CD71, (5) IRP1, (6) IRP2 and (7) GAPDH (*n*=4). (**H**) HEY cells were treated as described in (A). Glutamate levels were assessed by harvesting the media supernatant at 24 h (left panel) or 48 h (right panel) following treatment (*n*=2).

IRP1, when bound to iron, is involved in the catalysis of citrate to iso-citrate, the first rate-limiting enzymatic step in the conversion of citrate into glutamate [1]. Of note, glutamate induces cell death in a similar manner to ferroptosis in a MAPK-dependent manner [11,28]. Therefore, we next investigated whether inhibition of aconitase activity, via the use of OMA (which targets cytosolic and mitochondrial aconitases [29–32]), reverses the FAC-mediated cell death response and restores survival in HEY cells. As shown in Figures 5A and 5B, OMA potently reversed cell death induced by FAC in HEY cells. Furthermore, the combinatorial action of OMA with FAC recovered TOM20 and TOM70 protein expression (Figure 5C). However, the increase in LC3-II levels induced by FAC was only subtly reduced by co-treatment with OMA (Figures 5C, 5D and 5E). The changes in lysosome numbers, assessed using LysoTracker Red, also appeared to be markedly less compared with FAC alone (Figure 5F).

We next assessed the cellular response of the cells treated with IRP1 and/or IRP2 siRNA in combination with 48 h FAC to assess whether reduced expression of IRP1 (which is associated with aconitase activity) and/or IRP2 could lead to altered expression of TOM proteins. However, we did not observed any marked changes in TOM20 or TOM70 (Figure 5G). Therefore, other mechanisms are likely accountable for the changes observed with OMA, possibly mitochondria related.

Since we earlier demonstrated that GSH levels were reduced in HEY cells treated with FAC, we quantified extracellularly secreted glutamate levels (measured in the media supernatant). The results indicate that there were no significant changes in glutamate levels upon FAC treatment (Figure 5H). However, glutamate levels in both OMA alone or in combination with FAC were significantly lower, suggesting that inhibition of aconitase activity can diminish extracellularly secreted glutamate in both untreated and FAC treated HEY cells.

## DISCUSSION

**Figure 6:**
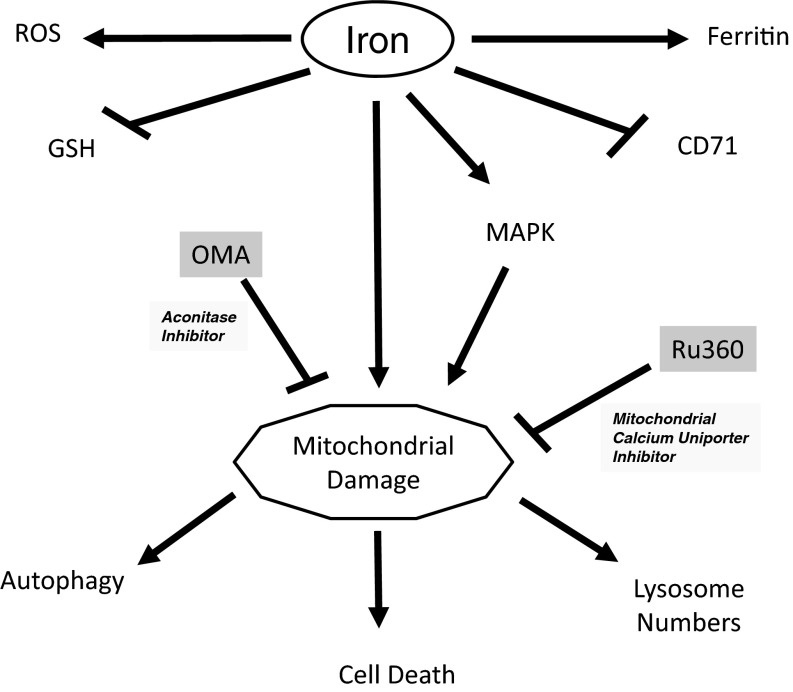
Involvement of mitochondria in FAC-mediated cell death in HEY cells Iron treatment in HEY cells leads to an imbalance in redox levels since ROS levels are increased and GSH (antioxidant) levels are decreased. Via IRP1/2 translational regulation of key targets involved in iron metabolism, FAC leads to increased ferritin and decreased CD71 protein levels. Iron leads to loss of OMM proteins (TOM20 and TOM70) in a MAPK and mitochondrial calcium uniporter-dependent manner. This was demonstrated via the use of U0126 (MAPK inhibitor) and Ru360 (MUC inhibitor). Activation of aconitase activity as well as increased mitochondrial damage contributes to cell death in HEY cells. Lysosomal and autophagic events are located downstream of the mitochondrial events.

Oxidative stress, induced by excess iron, is an event which involves increased radical production (in both the cytosolic and mitochondrial compartments) [33,34], which, together with an imbalance in antioxidant defense pathways, leads to cell damage and death [18]. We have summarized our findings in Figure 6. Herein, we show that iron (presented as non-transferrin bound iron, a form commonly increased in patients with iron-overload conditions such as that present in endometriotic cysts [10,23], a precursor lesion thought to lead to development of ovarian cancer subtypes) treatment in multiple ovarian cell lines leads to increased ROS levels, detected using H_2_DCFDA. Although increased ROS can promote damage by modifying cellular constituents (lipids, proteins, as well as DNA mutations) leading to cell death [18], we did not identify differences in ROS levels among the cell lines tested with respect to iron-induced reduction in cellular viability. Activation of cellular defense mechanisms may antagonize ROS production to protect cells from increased ROS [33,34]. Interestingly, in the serous ovarian carcinoma HEY cell line (which contains a Ras mutation and undergoes apoptosis/necrosis in response to FAC) [7], GSH levels were significantly reduced with iron treatment. In contrast, GSH levels remained unchanged in the T80 cell line treated with FAC; indeed, we previously reported that T80 cells do not undergo cell death following iron treatment. Thus, the redox environment between T80 and HEY cells appears different and may be responsible for the differences in cell survival in response to FAC. However, our attempts to reverse the ROS generation via the use of NAC or catalase failed (results not shown); indeed, catalase has been reported to increase the cell death response in cells [35].

Internalization of CD71 when bound to transferrin-iron complex increases intracellular iron levels [2,3]. Iron in the labile iron pool binds to IRP1 (as well as IRP2) to regulate the translation of specific targets including FTH and CD71 mRNA [2,3]. Although there are multiple reports implicating their levels in regulating the cellular response to iron [36–39], we did not identify any differences between their protein levels in our iron-sensitive (HEY, TOV21G and T80+H-Ras) and iron-resistant (T80 and TOV112D) cell lines. Interestingly, in the presence of high iron, IRP1 dissociates from iron-responsive elements (IRE) and elicits cytosolic aconitase activity [1]. Although FAC treatment did not increase extracellular levels of glutamate (via the use of OMA, a competitive inhibitor of aconitase), OMA did reduce glutamate levels and reverse the cell death response in HEY cells.

The cellular response to glutamate appears similar to the recently described ferroptotic-mediated cell death event [11]. Both processes involve the inhibition of cystine uptake through the cystine/glutamate antiporter (system x_c_^−^) leading to reduced GSH antioxidant levels and increased cell death [11]. Both cell death mechanisms involve aberrations in the mitochondria and activation of the MAPK pathway [11,28,40]; furthermore, both cell death mechanisms can be inhibited by ferrostatin-1 [11]. Therefore, there are multiple similarities between glutamate-induced cell death and ferroptosis. In our studies, although iron modulated the MAPK pathway and reduced GSH levels while increasing ROS in the HEY cell line (relative to the T80 cell line), our earlier studies with ferrostatin-1 did not demonstrate iron-induced cell death response [7]. Moreover, we observed changes in the autophagic response while the ferroptosis/glutamate-induced cellular events appear to be independent of changes in autophagy [11]. Although we noted changes in FTH and CD71 upon FAC treatment, IRP1 and/or IRP2 knockdown did not modulate iron-sensitivity in our HEY cell line, which contrasts with that described in ferroptosis [11]. Although the mitochondria were smaller (with increased membrane density) in ferroptotic cells [11], we noted marked changes in mitochondrial morphology and consumption by autophagosomal membrane.

Mitochondria elicit multiple critical cellular functions including (1) energy generation via the electron transport chain, (2) heme synthesis, (3) iron–sulfur cluster biogenesis and (4) iron metabolism [12]. For these functions, mitochondrial iron import mechanisms are essential. Specific proteins involved in mitochondrial iron transport include mitoferrins [located in the inner mitochondrial membrane (IMM)] [12] as well as the MCU which appears to contribute to iron import (via studies using Ru360, an inhibitor of this channel) [14,15,25,27]. Indeed, communication between the cytosolic and mitochondrial compartments is critical for regulation of iron metabolism; these processes are dysregulated in a variety of diseases including Friedreich's ataxia [41,42]. From our TEM analysis of FAC-treated HEY cells, we observed both damaged mitochondria as well as their apparent consumption by autophagosomal membrane (i.e. mitophagy). In addition, we observed a marked reduction in specific OMM proteins (i.e. TOM20 and TOM70). Recent studies have observed PINK1 (a serine-threonine kinase) to stabilize on the OMM, thus promoting the recruitment of Parkin (an E3 ubiquitin ligase) which ubiquitinates and degrades specific targets on the OMM including TOM20, TOM40 TOM70 (in addition to voltage-dependent anion channel (VDAC), mitofusin, Bcl-2 and Drp1); this event leads to mitochondrial rupture and loss of membrane permeability [43–45]. These damaged mitochondria are then recognized by the autophagosomal machinery and degraded within the autophagosome in the process of mitophagy. In contrast with OMM proteins, the degradation of the IMM components is independent of PINK1 and Parkin but dependent on the process of mitophagy [46,47].

We previously demonstrated that FAC treatment in T80 cells dramatically increases lysosome numbers (via TEM analysis) [7]. We now report cellular changes by TEM in the HEY cell line which undergoes apoptosis/necrosis upon FAC treatment. We noted that HEY cells which were necrotic clearly lacked lysosomes, whereas cells which were viable but stressed had large abundant lysosomes. Since iron recycling occurs mostly within lysosomes, and these organelles contain high levels of redox-active iron [48,49], the lysosomes may be susceptible to LMP and rupture. In addition, we demonstrate cross-talk between the lysosomal and mitochondrial compartments following iron treatment. Although we noted that inhibition of the MCU using Ru360 markedly increased lysosome size and abundance, suggesting that the mitochondrial changes occur upstream of changes in the lysosome, existing literature suggests that lysosomal changes precede mitochondrial changes (hydrolases released from lysosomes is suggested to promote mitochondrial damage) [50–54]. Furthermore, we noted a reduction in LC3-II and GFP-LC3 punctae upon Ru360 treatment in FAC treated cells; this result suggests that the autophagic changes are likely to be events involving degradation of the mitochondria (i.e. mitophagy).

## Online data

Supplementary data
