# Supplementary material for: Iron alters cell survival in a mitochondria-dependent pathway in ovarian cancer cells
Source: Biochem J. 2015 Feb 20;466(Pt 2):401–13. doi: 10.1042/BJ20140878 (PMC4338747; doi:10.1042/BJ20140878)
Supplement: Supplementary data [file bj4660401ntsadd.pdf]

Supplementary Figure Legends

Figure S1 A, HEY cells were seeded at 325,000 cells in each well of a 6-well plate. Following overnight adherence, cells were treated with non-targeting, TOM20, or TOM70 siRNA on two successive days as described in Experimental section. This was followed by treatment with FAC (250 M) for 48 hours. Cell lysates were then analyzed by western blotting using the following antibodies: (1) LC3B, (2) TOM20, (3) TOM70, and (4) GAPDH (n=2). B, HEY cells were seeded on glass coverslips in each well of a 6-well plate. Following attachment, cells were treated with non-targeting or TOM70 siRNA on two successive days. Next, the cells were treated with FAC (250 M) for 48 hours. Cells were then viewed using an inverted fluorescence microscope and a 60X oil immersion objective. Representative images are shown (n=4). C, Cells were treated as described in B with the addition of LysoTracker Red for 1 hour. Cells were stained with DAPI and imaged using an inverted fluorescence microscope and a 60X oil immersion objective. Representative images are shown (n=4).

Figure S2 Left panel, HEY cells were seeded at 250,000 cells in each well of a 6 well plate. Following cell adherence, cells were treated with H2DCFDA for 30 minutes prior to addition of FAC (250 M) or H2O2 (100 M). Media supernatant and adherent cells were collected following 24 hour treatment into PBS. ROS were measured via flow cytometry (n=2). Right panel, HEY cells were seeded at 250,000 cells in each well of a 6-well plate. Following adherence, cells were treated with H2DCFDA for 30 minutes prior to addition of FAC (250 M) alone, Ru360 (10 M) alone, U0126 (10 M) alone, Ru360 (10 M) in combination with FAC (250 M), and U0126 (10 M) in combination with (250 M). Media supernatant and adherent cells were collected following 24 hour treatment into PBS. ROS were measured via flow cytometry (n=2).

Figure S1

A

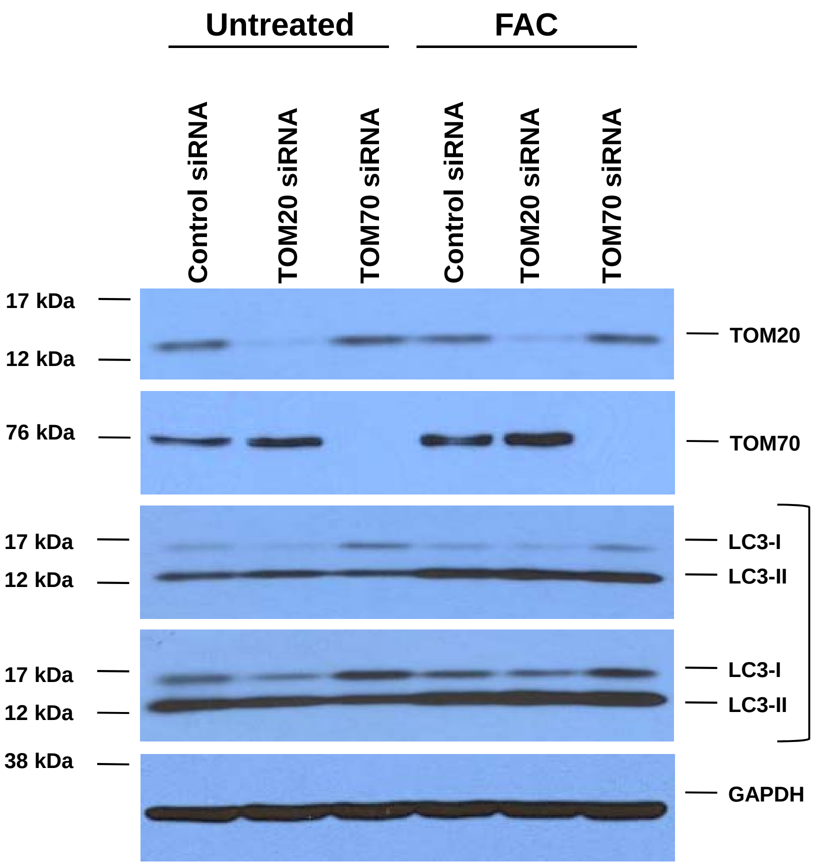

Untreated
FAC
Control siRNA
TOM20 siRNA
TOM70 siRNA
Control siRNA
TOM20 siRNA
TOM70 siRNA
17 kDa
12 kDa
TOM20
76 kDa
TOM70
17 kDa
12 kDa
LC3-I
LC3-II
17 kDa
12 kDa
LC3-I
LC3-II
38 kDa
GAPDH

Figure S1

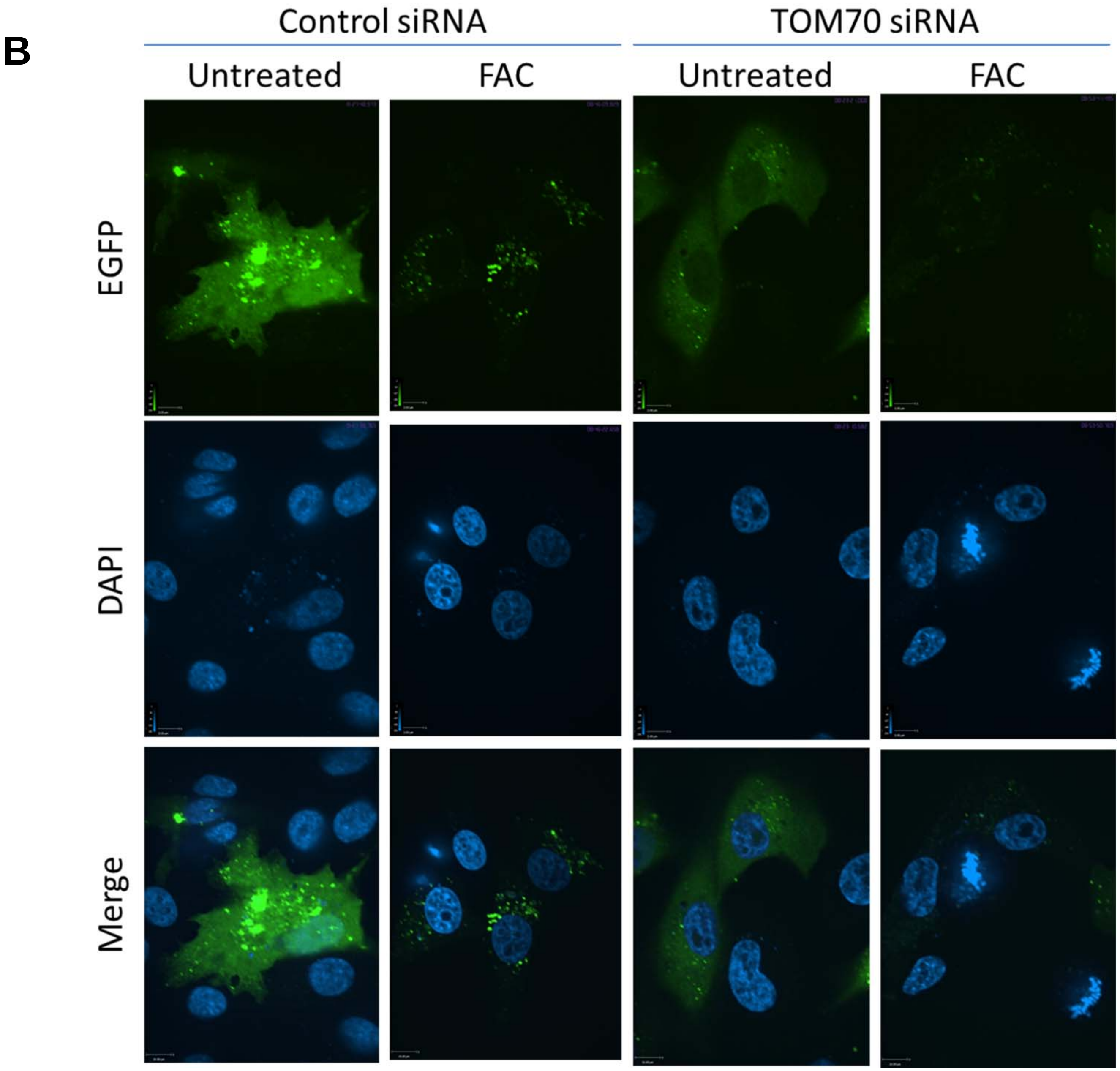
B
Control siRNA
TOM70 siRNA
Untreated
FAC
Untreated
FAC
EGFP
DAPI
Merge

Figure S1

C

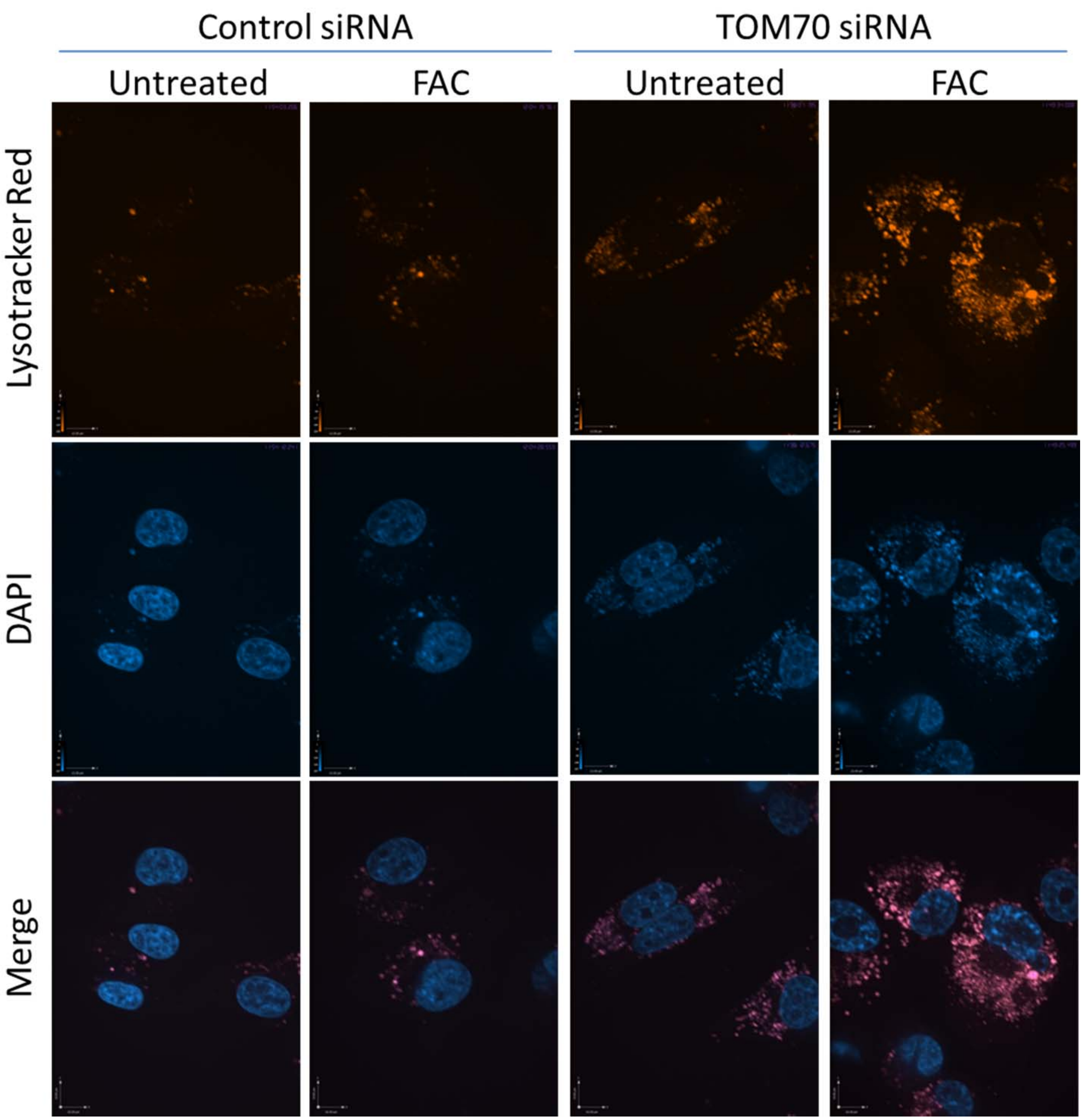

Control siRNA
TOM70 siRNA
Untreated
FAC
Untreated
FAC
Lysotracker Red
DAPI
Merge

Figure S2

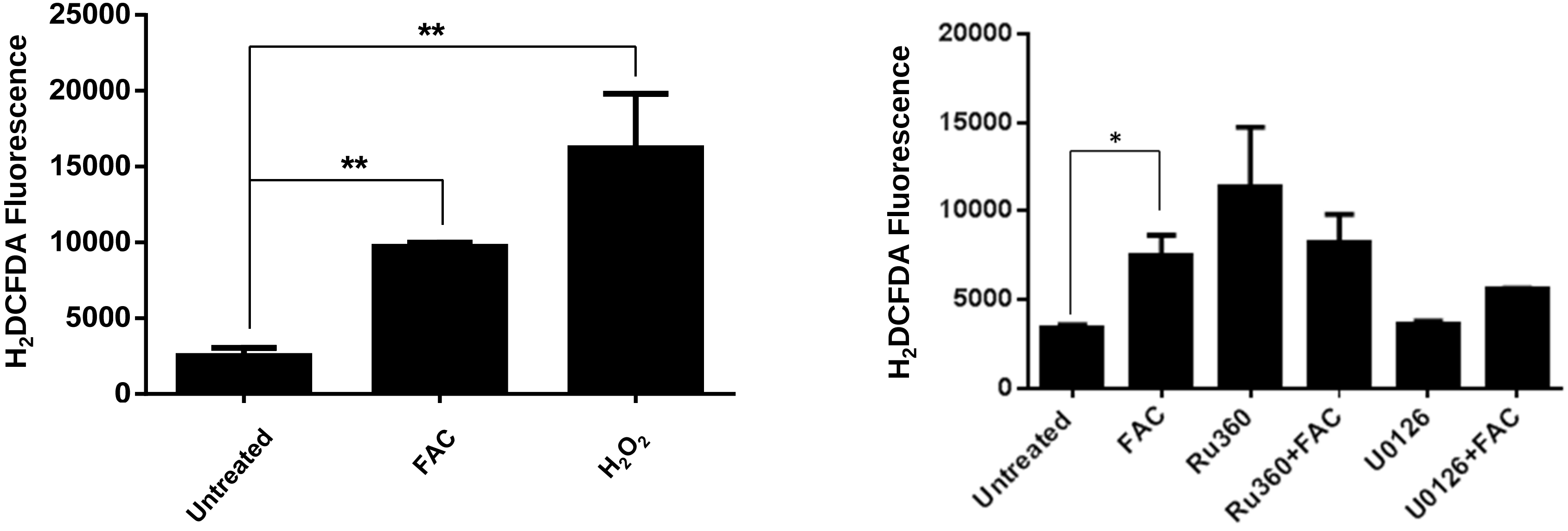

H2DCFDA Fluorescence
25000
20000
15000
10000
5000
0
**
**
Untreated
FAC
H2O2
H2DCFDA Fluorescence
20000
15000
10000
5000
0
*
Untreated
FAC
Ru360
Ru360+FAC
U0126
U0126+FAC
